# Coexistence of multiple minor states of fatty acid binding protein and their functional relevance

**DOI:** 10.1038/srep34171

**Published:** 2016-09-28

**Authors:** Binhan Yu, Daiwen Yang

**Affiliations:** 1 Department of Biological Sciences, National University of Singapore, 14 Science Drive 4, 117543, Singapore

## Abstract

Proteins are dynamic over a wide range of timescales, but determining the number of distinct dynamic processes and identifying functionally relevant dynamics are still challenging. Here we present the study on human intestinal fatty acid binding protein (hIFABP) using a novel analysis of ^15^N relaxation dispersion (RD) and chemical shift saturation transfer (CEST) experiments. Through combined analysis of the two types of experiments, we found that hIFABP exists in a four-state equilibrium in which three minor states interconvert directly with the major state. According to conversion rates from the major “closed” state to minor states, these minor states are irrelevant to the function of fatty acid transport. Based on chemical shifts of the minor states which could not be determined from RD data alone but were extracted from a combined analysis of RD and CEST data, we found that all the minor states are native-like. This conclusion is further supported by hydrogen-deuterium exchange experiments. Direct conversions between the native state and native-like intermediate states may suggest parallel multitrack unfolding/folding pathways of hIFABP. Moreover, hydrogen-deuterium exchange data indicate the existence of another locally unfolded minor state that is relevant to the fatty acid entry process.

In order to perform their biological functions, proteins need not only to maintain correct tertiary structures but also to change the structures to appropriate conformations under various conditions^1^. In either native or non-native environments, in principle a protein can access all conformations or has multiple conformations in a dynamic equilibrium manner. The ground state is often observable directly by biophysical techniques, but the “excited states” are “invisible” to most experimental approaches due to their low populations. Nuclear magnetic resonance (NMR) is one of a very few techniques with which the minor “excited” states can be visible indirectly and then characterized in details^2^,^3^,^4^,^5^,^6^,^7^, when their lifetimes are in the range of sub-seconds to microseconds. Characterizing such “excited” states can provide rich information that is crucial for solving the riddles of how proteins fold to their correct tertiary structures and interact with their specific binding partners^8^,^9^,^10^. Currently, studies on the “excited” states or minor states by NMR, especially under physiological conditions, are often limited to two- or three-state exchange scenarios^2^,^3^,^7^. This is because the observables obtained with one experimental approach are sensitive only to certain states with lifetimes in a relatively narrow range of timescales and the observables have a complex relation to the number, populations, and chemical shifts of truly existing minor states as well as the conversion rates among different states^3^. To better characterize the minor states, collective application of different experiments that are complementary in timescales becomes a viable choice.

Herein we present a dynamics study of human intestinal fatty acid binding protein (hIFABP) using a novel analysis of ^15^N relaxation dispersion (RD) and chemical exchange saturation transfer (CEST) experiments. FABPs (~15 *kD*) are cytosolic proteins with high binding affinity to long chain fatty acids. They have highly conserved structures with a slightly elliptical β barrel comprising 10 antiparallel β strands and a cap consisting of two short α helices (Fig. 1). Earlier dynamics studies on rat IFABP suggested the second helix, the turn between β-strands C and D, and the turn between strands E and F as the putative portal region for ligand entry/exit^11^,^12^ since they are more flexible on ps-ns and ms-μs timescales than other regions. Subsequent studies have shown that the ps-ns and ms dynamics are irrelevant to the ligand entry process^13^,^14^. Very recently using a cap-closed variant of hIFABP in which the helical cap is locked to the barrel through a disulfide linkage, we have demonstrated that local unfolding of the second helix provides an opening for ligand entry and the lifetime of the locally unfolded state is about 70 μs^6^. In this work based on RD and CEST data, we found that wild type (WT) hIFABP in the absence of ligands exists in four conformational states: one major state and three minor intermediate states. The three minor states are native-like and each interconvert with the major state with exchange rates ranging from 36 s^−1^ to 3086 s^−1^, suggesting the presence of multiple independent protein folding pathways. In addition, a locally unfolded state also likely exists in WT hIFABP since the hydrogen-deuterium exchange protection factors of the amides in the second helix are smaller than 100 and those of V17-M21 in the first helix are larger than 10000.

## Results

**Figure 1:**
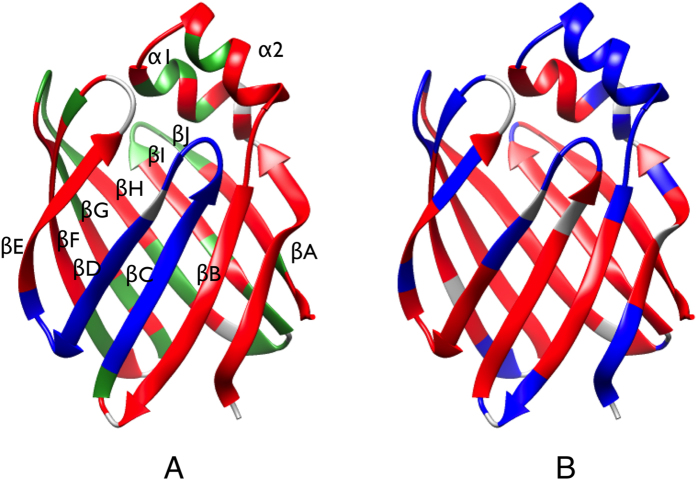
(**A**) Distribution of residues displaying three conformational states (red), four states (blue), and no obvious relaxation dispersion (green). (**B**) Distribution of residues displaying amide hydrogen exchange protection factors larger than 100 (red) and smaller than 100 (blue). The residues without available data are colored in grey.

**Figure 2:**
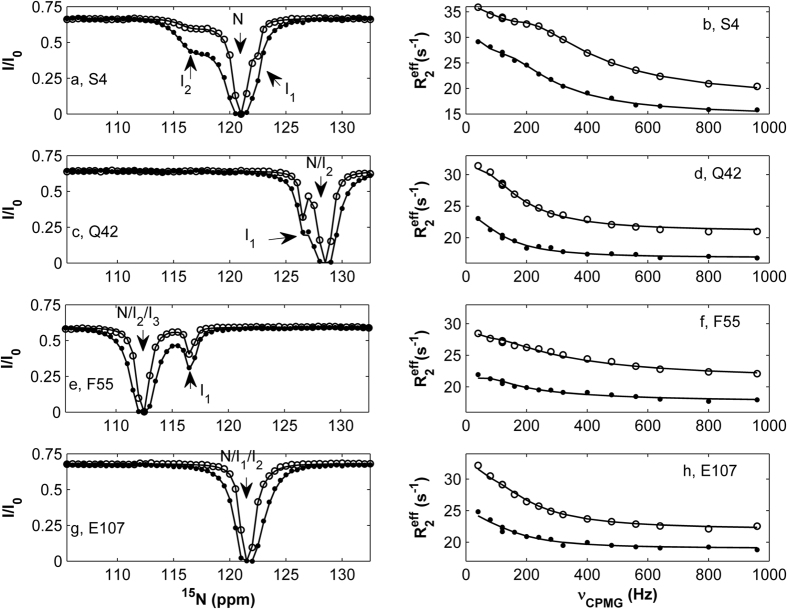
Representative CEST (**a**,**c**,**e**,**g**) and RD (**b**,**d**,**f**,**h**) profiles. The experimental CEST data at rf fields of 13.6 and 27.2 Hz are indicated by “o” and “●”, respectively. The experimental RD data at 800 and 500 MHz are indicated by “o” and “●”, respectively. The solid lines are best fits obtained with model I (**a–d, g,h**) and model IV (**e,f**). The locations (or chemical shifts) of states N, I1, I2, and I3 in the CEST profiles are indicated by arrows.

One hundred and five ^15^N-^1^H HSQC peaks from 130 backbone amides were well resolved in the spectra of hIFABP recorded on both 500 and 800 MHz NMR spectrometers. ^15^N CEST and RD data were analyzed for determining the number of conformational states and kinetic parameters. Out of 131 residues in hIFABP, one residue (Ser4) showed one major dip and two minor dips in its CEST profiles (Fig. 2a), while 43 each displayed one major dip and one minor dip (Fig. 2c,e). Moreover, 62 and 89 residues displayed obvious relaxation dispersion with exchange contributions to transverse relaxation rates (R_ex_) larger than 2 s^−1^ at 500 and 800 MHz spectrometers, respectively (Fig. 2b,d,f,h). The data indicate that most residues or all the secondary structure elements (α helices and β strands) of hIFABP undergo slow conformational exchanges between the observed major state and “invisible” minor states on the millisecond timescale (Fig. 1A). Here a protein state is defined as a conformation with a unique set of ^15^N chemical shifts. Two different chemical shift states correspond to two distinct conformations which may have identical free energy.

When the CEST profiles with two dips (one major and one minor dip) separated by ≥160 Hz were fitted individually to a two-state exchange model (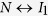, where N and I_1_ represent native and minor states, respectively), the resultant average exchange rate (k_ex1_) between N and I_1_ states and average population of state I_1_ (P_I1_) were about 60 s^−1^ and 3%, respectively. If there existed only one global exchange process in hIFABP, the R_ex_ values for those residues used in the CEST fitting would be smaller than 2 s^−1^. In fact, they were significantly larger than 2 s^−1^ (Fig. 2b,d,f,h), demonstrating the existence of at least one more conformational exchange between state N and another minor state (I_2_). The presence of two minor states is also evident from the two minor dips in the CEST profile of Ser4 (Fig. 2a).

When the RD data of the same set of residues used in the CEST fitting were fitted individually to a two-state exchange model (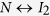), the resultant average k_ex2_ values were about 600 s^−1^, which is much larger than that obtained from the CEST data, and the resultant chemical shifts of state I_2_ were very different from those of I_1_. Therefore, two minor states coexist with a major native state (N) and they undergo conformational exchanges with state N at very different rates. Except for Ser4, state I_2_ was unobservable for all other residues by the ^15^N CEST experiment because its exchange rate with state N is significantly larger than the resonant frequency differences of ^15^N spins in states N and I_2_ (i.e., k_ex2_ > 2π[ν(N) – ν(I_2_)]).

In order to extract kinetic parameters for the two exchange processes, we analyzed the data of the residues displaying two and three obvious CEST dips (separated by >80 Hz) and also having R_ex_ values larger than 2 s^−1^ at the 500 MHz NMR spectrometer. In total, 44 residues met the requirements. For each of these residues, the CEST data together with the RD data were fitted to a three-state model (model I):


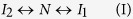


The resultant (k_ex1_, P_I1_) values were quite uniform (~40 s^−1^, ~4%) for all the 44 residues. Differently, (k_ex2_, P_I2_) values distributed in two distinct regions: one centered at (~850 s^−1^, ~2%) for 29 residues and the other at (~1200 s^−1^, ~4%) for the rest 15 residues. These 15 residues (Phe47, Thr48, Lys50, Glu51, Ser52, Ser53, Ala54, Phe55, Arg56, Ile58, Glu59, Vla60, Phe62, Leu64, Vla66) are located at β-strands C (Lys46 – S53) and D (Ile58 – Phe62), loop between βC and βD (Ala54 – 57), and loop between βD and βE (63–66). Note that Val66 is in a loop in most hIFABP structures deposited in PDB, but it is at the beginning of βE in the NMR structure of WT hIFABP in the absence of ligands (PDB code: 3IFB). The region from Phe46 to Val66 is denoted here as βC&βD, which forms a part of the so-called gap region. Therefore, the 44 sets of data from 44 residues were divided into two groups: G1 with 29 residues located outside βC&βD and G2 with 15 residues inside βC&βD.

Subsequently, all the CEST and RD data for G1 were fitted simultaneously to model I. The total fitting residual (χ^2^) was 6369 and reduced χ^2^ (χ_red_^2^) was 1.06. Both the CEST and RD data agree very well with the fits (Fig. 2 and Fig. S1). The resultant kinetic parameters were k_ex1_ = 36 ± 5 s^−1^, k_ex2_ = 832 ± 10 s^−1^, p_I1_ = 4.3 ± 0.4%, and p_I2_ = 2.09 ± 0.02%. The derived chemical shifts in states I_1_ (δ_I1_) and I_2_ (δ_I2_) are listed in Table S1. In the same manner, the data for G2 produced the following result: χ^2^ = 6010, χ_red_^2^ = 1.80, k_ex1_ = 38 ± 4 s^−1^, k_ex2_ = 1174 ± 83 s^−1^, p_I1_ = 4.1 ± 0.3%, and p_I2_ = 3.8 ± 0.3%. Although the CEST data agree well with the fits, the RD data do not agree well (Fig. S2), indicating that model I is not good enough for G2. The experimental errors for G1 and G2 were similar, but the χ^2^_red_ value for G2 is much larger than that for G1, further supporting that model I is not suitable to G2. We also tested the other two possible three-state models (model II and III):


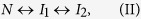



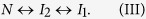


When model II was used, χ^2^ = 8113 and χ_red_^2^ = 1.34 for G1; χ^2^ = 6493 and χ_red_^2^ = 2.00 for G2. F-test analyses show that model II can be rejected at confidence intervals of >99.999% and 95% for G1 and G2, respectively. When model III was used, neither G1 nor G2 data could be fitted. Therefore, except the βC&βD region, hIFABP can be described very well by model I in terms of conformational exchanges.

To assess the necessity of combining both CEST and RD data, we analyzed the CEST and RD data from G1 separately. When the CEST data were fitted to a global two-state model, the extracted k_ex_ and P_I_ values were 71 ± 14 s^−1^ and 2.8 ± 0.7%, respectively, and χ^2^ = 19243. The χ^2^ value is much larger than that obtained with model I, indicating bad fitting. The kinetic parameters obtained are significantly different from those for the slower exchange process derived from model I using both CEST and RD data (36 s^−1^, 4.3%). Although CEST is sensitive to slow conformational exchange, its profile is also affected by intermediate exchanges (k_ex_ ≤ 6Δω, where Δω is the angular frequency difference between the minor and major states). So it is not surprising to obtain inaccurate results by neglecting the intermediate exchange process. When the RD data were fitted to a global two-state model, the extracted k_ex_ and P_I_ values were 593 ± 25 s^−1^ and 2.8 ± 0.5%, respectively, and χ^2^ = 4912. The results are also significantly different from those for the faster exchange process derived from model I (832 s^−1^, 2.09%). The discrepancies result from ignoring the contribution of the slow exchange process to the RD profiles. Therefore, it is necessary to use both CEST and RD data to obtain reliable kinetic parameters.

For a well folded protein, the motions on ms – μs timescales, either regional or global, are often collective. This means that the residues located in the same area are bound to move in a cooperative way. Since k_ex1_ and P_I1_ values obtained from G_1_ and G_2_ are nearly identical, the conformational exchange between states N and I_1_ should be a global process for the entire protein. Because the k_ex2_ and P_I2_ values for G1 are significantly different from those for G2, βC&βD must behave differently from the rest part of the protein. One possibility is that the two parts move in an uncorrelated manner in state I_2_. A more likely scenario is the presence of one more minor state, state I_3_, since a three-state model is not good enough to describe the conformational exchanges of βC&βD. In this case, we assumed that all hIFABP residues undergo two global conformational exchanges, one between states N and I_1_ and the other between states N and I_2_, and that the residues in βC&βD experience a local conformational exchange between states N and I_3_ (model IV):


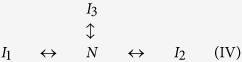


By fixing k_ex1_, p_I1_, k_ex2_, and p_I2_ at the values derived from G1, we fitted the G2 data to model IV. The obtained parameters were χ^2^ = 3818, χ_red_^2^ = 1.14, k_ex3_ = 3086 ± 363 s^−1^, and p_I3_ = 1.2 ± 0.2%. The fitting with model IV is much better than that with model I (Fig. S2). An F-test analysis also showed that the fitting using the 4-state model gives significant improvement (p < 10^−10^) over the 3-state model. Selection of model IV rather than model I is further supported by the χ_red_^2^ values (1.14 vs. 1.80). Therefore, the conformational exchanges of βC&βD region can be described by model IV. The derived chemical shifts of ^15^N spins in state I_3_ are listed in Table S1. Note that k_ex3_ is close to 2π[ν(N) – ν(I_3_)] for Val60 (Table S1) or the exchange between states N and I_3_ is not in the fast exchange regime for Val60. Thus p_I3_ and the chemical shifts of state I_3_ could be well determined independently.

Besides the 44 residues described above, 45 residues each had a RD value larger than 2 s^−1^ at 800 MHz but showed only one apparent CEST dip (Fig. 2g). 41 out of the 45 residues are located outside the βC&βD region, and their chemical shifts in states I_1_ and I_2_ were extracted with model I by fixing k_ex1_, p_I1_, k_ex2_, and p_I2_ at the values derived from G1. The rest 4 residues are located inside the βC&βD region, and their chemical shifts in states I_1_, I_2_, and I_3_ were extracted with model IV by fixing k_ex1_, p_I1_, k_ex2_, p_I2_, k_ex3_, and p_I3_ at the values derived from G2. The obtained chemical shifts are listed in Table S1.

To examine if the conformational exchanges observed here are caused by protein self-association (or oligomerization), we also conducted the experiments at a significantly lower protein concentration (0.7 mM). Both the CEST and RD profiles were similar to those obtained at a higher concentration (1.5 mM) (Fig. 1 and Fig. S3). According to the CEST profile of S4 (Fig. S3a), two minor states are visible directly, same as that at the high concentration. Also, the data could be divided into two groups: G1 and G2. Fitting the G1 data to Model I, we obtained k_ex1_ = 38 ± 4 s^−1^, k_ex2_ = 837 ± 10 s^−1^, p_I1_ = 4.6 ± 0.4%, and p_I2_ = 2.27 ± 0.05%. The G2 data could not be fitted well to model I. When the data were fitted to model IV by fixing k_ex1_, p_I1_, k_ex2_, and p_I2_ at the values derived from the G1 data, we obtained k_ex2_ = 2946 ± 350 s^−1^ and p_I1_ = 1.0 ± 0.2%. The kinetic parameters derived from the two samples at very different concentrations are nearly identical, indicating that all the exchanges observed are the intrinsic conformational changes rather than from the protein association-dissociation process.

**Figure 3:**
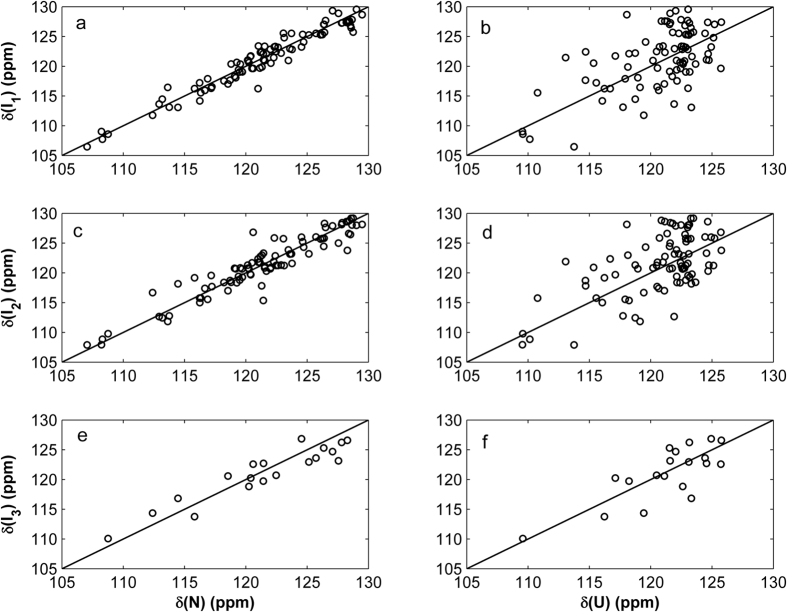
Comparison of chemical shifts of native state N with intermediate states I_1_ (**a**) I_2_ (**c**) and I_3_ (**e**) and comparison of chemical shifts of unfolded state U with states I_1_ (**b**), I_2_ (**d**), and I_3_ (**f**).

Figure 3 shows ^15^N chemical shift comparisons between the native and intermediate states and between the disordered (or unfolded) state and intermediate states. According to our previous work on the hIFABP variant^6^, we found that the differences between the predicted ^15^N shifts in the unfolded state and the experimental shifts measured at 8 M urea are smaller than 1 ppm for more than 85% residues. So the predicted shifts used here can be considered as good approximations of the true shifts in an unfolded state. The significant discrepancy for most residues observed in Fig. 3b,d,f (> 1 ppm) should not result from the approximations. In terms of chemical shifts, the minor states (I_1_, I_2_, and I_3_) are much more close to the native state than the unfolded state. Because backbone ^15^N chemical shifts are influenced by not only secondary structure but also hydrogen bonding and charges, we are unable to determine the secondary structure of the intermediate states. Nevertheless, chemical shift resemblance between the native and intermediate states indicates that all the three minor states are native-like instead of unfolded-like.

To further characterize the structural feature of the minor intermediate states, we performed hydrogen-deuterium exchange (HDX) experiments. 49 residues were unobservable in the first HDX spectrum because their HDX rates were larger than 0.01 s^−1^ and the exchange with deuterium was nearly completed within the dead time (~180 s). Most of these residues are not involved in H-bonding between backbone N-H and O=C moieties. They are located in loops, strands βD and βE, and beginning of helices. Although the amides of residues 27–31 in helix 2 are involved in H-bonding in the native state, they were not protected from HDX. This may be caused by local unfolding of this helix as shown very recently by using a hIFABP variant with a disulfide linkage between helix 2 and loop βE-βF^6^. For the residues with fast HDX rates (>0.1 s^−1^), their amide hydrogen exchange rates were measured on a sample in 95% H_2_O. The derived exchange rates and protection factors (P) are summarized in Table S1. We have shown that the amide hydrogen exchanges occur through an EX2 limit for nearly all residues of the hIFABP variant^6^. Since the WT hIFABP and its variant have very similar structures, the EX2 condition should also be applied to the WT protein. Under EX2, the population of the “open” state (P_O_) can be approximated as 1/P (P_O_ ≈ 1/P). If states I_1_, I_2_, and I_3_ were entirely unfolded or all the amides are in an “open” state, the observed P values would be smaller than 100 for all amides since the populations of the three minor states are all larger than 1%. In fact, many residues located outside and inside the βC&βD region had P values much larger than 100 (Fig. 1B). Therefore, states I_1_, I_2_, and I_3_ are not unfolded and should be native-like, which is consistent with the conclusion drawn from the chemical shifts.

## Discussion

According to RD data alone, one can determine if conformational exchange exists in a protein but cannot directly tell the number of minor states involved in the exchange process^3^. Using statistical approaches, one can identify which conformational exchange model fits well to the experimental data and then determine the number of conformational states^4^,^6^. However, it is difficult to go beyond a 3-state model from the RD data analysis in the absence of other experimental data. Moreover, absolute values of chemical shift differences between a minor state and a major state rather than the chemical shifts of a minor state are determined from RD data^3^. To determine the structure of a minor state, it is preferable to have the chemical shifts. Currently, the chemical shifts are determined from the absolute values and their signs which can be obtained in the case where one minor state exists by comparing HSQC or HMQC spectra recorded at multiple magnetic fields^15^. If two or more minor states coexist, the signs will be difficult to be determined.

According to CEST data alone, one can see directly the number of minor states and their chemical shifts without involvement of any fitting models^16^,^17^. At first glance it seems that CEST is superior to RD in characterizing protein conformational exchanges. In fact, the minor states directly observed in a CEST profile can be a subset of truly existing states in a protein because the states in fast and intermediate exchange regimes are not observable by CEST^18^. This argument is also supported by our results – only up to two minor states were observed in the^15^N CEST profiles although at least three minor conformational states exist in hIFABP.

When both CEST and RD data are used simultaneously, the number of minor states existing in a protein and the kinetic parameters can be better determined in comparison with use of either CEST or RD data because CEST covers a relatively slow exchange regime while RD covers intermediate and relatively fast exchange regimes. Our results indicate that the coverage in exchange rates is from ~30 s^−1^ to ~3000 s^−1^ when ^15^N CEST and RD data are used together. In order to probe faster exchange processes, one can use ^1^H CPMG RD^6^ or ^15^N off-resonance R_1ρ_ RD^19^.

When a minor state is unobservable directly in the CEST profile of a ^15^N spin but this state contributes to the RD of the same ^15^N spin, the CEST major dip is asymmetric (Fig. 2g) and the asymmetry depends on the sign of the chemical shift difference between the major and minor states. So the sign can be determined when CEST and RD are analyzed together. For a given residue, the fitting residual (χ^2^) for the case where the sign of the chemical shift difference is correct should be significantly smaller than that for the case where the sign is wrong. Here we use E107 (which displays only one major dip, Fig. 2g) as an example to illustrate how sensitive the χ^2^ is to the signs of chemical shift differences. When ν(I_1_) – ν(N) = −0.41 ppm and ν(I_2_) – ν(N) = −1.83 ppm, χ^2^ values for the CEST and RD data were 3025 and 79, respectively, the CEST data did not fit well to the model (Fig. S4). When ν(I_1_) – ν(N) = +0.41 ppm and ν(I_2_) – ν(N) = −1.83 ppm, similar large χ^2^ values were obtained. When ν(I_1_) – ν(N) = −0.41 ppm and ν(I_2_) – ν(N) = +1.83 ppm, χ^2^ values for the CEST and RD data were 62 and 71, respectively, and the CEST data fitted very well to the model (Fig. S5). When ν(I_1_) – ν(N) = +0.41 ppm and ν(I_2_) – ν(N) = +1.83 ppm, χ^2^ values for the CEST and RD data were 205 and 79, respectively. By visual inspection, the fitting curve with a value of −0.41 ppm for ν(I_1_) – ν(N) is slightly better than that with a value of +0.41 (Fig. S6). On the basis of χ^2^ values and F-test, ν(I_1_) – ν(N) should be −0.41 ppm. According to our hIFABP data, we could determine the signs of ν(I_1_) – ν(N), ν(I_2_) – ν(N), and ν(I_3_) – ν(N) when their absolute values are larger than 0.3, 0.5, and 1.0 ppm, respectively. To our knowledge, a combined analysis of both CEST and RD is used for the first time to determine the signs of ν(I) – ν(N) which are not available from RD alone.

Two major mechanisms have been evolved to describe protein-ligand binding processes: conformational selection (CS)^20^ and induced fit (IF)^21^,^22^. In conformational selection, a ligand selectively binds to a pre-existing species that is sparsely populated as an “active state” in equilibrium with the major ground inactive state. In the case of induced fit, a ligand interacts with the major species as to promote certain conformational changes followed by subsequent binding and eventually the formation of a ligand-protein complex. In principle, the binding mechanism can be assessed by tracking transient states with kinetics measurements. Structural studies of FABPs have shown that neither the ligand-bound *holo*-form nor the ligand-free *apo*-form displays obvious openings that are required for the ligand to access the internalized binding cavity, thus necessitating investigation of the intermediate states. According to k_ex_ and p_I_ values, the respective conversion rates from the native state to intermediates I_1_, I_2_ and I_3_ are 1.5, 17, and 38 s^−1^, much smaller than the rate limit of ligand binding to WT hIFABP as determined in our recent work (~1100 s^−1^)^6^. If the conversion from the native state to any one of the three intermediate states corresponded to the rate-limiting step before ligand binding, the conversion rate from the native state to the intermediate state would be similar to the rate limit. The disagreement between the conversion rate and the maximal ligand association rate indicates that the conformational exchanges described above are irrelevant to the ligand entry process.

Very recently using a hIFABP variant with a disulfide linkage between helix 2 and loop βE-βF, we have slowed down the ligand association rate limit from ~1100 to ~280 s^−1^ at 20 °C. Using this variant, we have identified a locally unfolded “open” minor state in which helix 2 is mainly unfolded. For the variant, the “open” state undergoes fast conformational exchange with the native “closed” state (~13500 s^−1^), and the conversion rate from the “closed” state to the “open” state (195 s^−1^) is consistent with the rate limit of fatty acid association (~180 s^−1^) at 13 °C, demonstrating this exchange process is functionally relevant. For WT hIFABP, we estimated that the exchange rate between the “open” state and the “closed” state should be larger than 75000 s^−1^ at 20 °C based on the maximal fatty acid binding rate (~1100 s^−1^) and by assuming that the WT hIFABP and its variant have similar “open” state populations (1.45%). Due to the low population of the “open” state and its fast exchange rate with the native state, the contribution of this state to RD could not be observed in the ^15^N CPMG RD experiments conducted here. Although the existence of the “open” state in WT hIFABP is not evident from our RD data, it can be deduced from our amide hydrogen exchange data. According to the structure of WT hIFABP, the backbone amides of V17-M21 (α1) and R28-A32 (α2) all form hydrogen bonds and have similar solvent accessibilities. If helices 1 and 2 behave similarly, they should have similar P factors. In fact, the P factors of V17-M21 (>10^4^) were much larger than those of R28-A32 (<10^2^) (Table S1), indicating that a small fraction of the second helix is in an “open” form. Therefore, hIFABP very likely uses a conformational selection mechanism to regulate the uptake and release of fatty acids.

Although states I_1_, I_2_, and I_3_ are irrelevant to fatty acid transport, they may play other functional roles since FABPs also interact with other proteins such as hormone-sensitive lipase and transcriptional factors^23^. To understand the functional relevance of these minor states, we have to further study the interactions of hIFABP with its binding partners. Interestingly, I_1_, I_2_, and I_3_ all are native-like and each interconvert with the native state (model IV). The exchanges observed here should come from true conformational conversions since hIFABP contains no proline residues and exists in a monomeric form under our NMR condition. Although no unfolded state was observed, the coexistence of multiple minor native-like states (I_1_, I_2_, and I_3_) each in direct conversion with the native state may represent a snapshot of the multitrack unfolding/folding processes^24^ in which each intermediate state occupies one of the parallel independent routes.

## Materials and Methods

### Sample preparation

^15^N labeled human intestinal fatty acid binding protein (hiFABP) was expressed and purified using a protocol described previously^14^. The NMR samples contained 20 mM sodium phosphate, 50 mM NaCl, 1 mM EDTA, 95% H_2_O, and 5% D_2_O. For NMR relaxation dispersion and CEST experiments, two samples were used: one with 1.5 mM and the other with 0.7 mM protein.

### NMR spectroscopy

Relaxation dispersion data on the sample with 1.5 mM protein were recorded on Bruker 800 MHz and 500 MHz spectrometers at 30 °C, using a pulse sequence described elsewhere^25^. A constant time delay (*T*_*CPMG*_ = 50 ms) was used with a series of CPMG field strengths (*ν*_*CPMG*_ = 40, 80, 120, 160, 200, 240, 280, 320, 400, 480, 560, 640, 800, 960 Hz). Each 2D data set comprised 640 × 100 complex points in the ^1^H and ^15^N dimensions and was recorded with 16 scans and inter-scan delay of 2 s. The experiments at *ν*_*CPMG*_ of 120 Hz were repeated three times for estimation of experimental uncertainties. If the uncertainties were smaller than 1%, an error of 1% was used in further data analyses. ^15^N CEST experiments^16^ were performed on a Bruker 800 MHz spectrometer at 30 °C with two weak radiofrequency (*rf*) fields of 13.6 and 27.2 Hz. For each *rf* field, 55 2D ^1^H-^15^N HSQC spectra were acquired with a series of ^15^N carrier frequencies ranging from 105.5 to 132.5 ppm at a spacing of 0.5 ppm. Each 2D data set comprised 640 × 100 complex points in the ^1^H and ^15^N dimensions and was recorded with 2 scans, an inter-scan delay of 1.5 s, and a saturation time (*T*_*EX*_) of 0.5 s. Reference spectra were also recorded with similar parameters except that *T*_*EX*_ = 0 s. For a given CEST profile, if the ^15^N chemical shift of the major state was larger than 119 ppm, the first 10 CEST data points from 105.5–110 ppm were used to estimate the uncertainty of this profile; otherwise, the last 10 points from 128–132.5 ppm were used. If the uncertainty was smaller than 1%, 1% was employed in further data analyses.

For the sample with 0.7 mM protein, RD experiments were done only on Bruker 800 MHz machine using a modified continuous wave decoupling CPMG scheme^26^. In this case, *T*_*CPMG*_ = 40 ms and *ν*_*CPMG*_ = 25, 50, 75, 100, 125, 150, 175, 200, 250, 300, 350, 400, 500, 600, 800, and 1000 Hz. To estimate errors, the experiments at *ν*_*CPMG*_ of 50 Hz were repeated two times. The number of scans of each FID for the RD and CEST data were 32 and 4, respectively. Other acquisition and processing parameters were the same for both samples.

HDX rates were measured by measuring the dependence of ^1^H-^15^N HSQC peak intensities on the time after dissolving a lyophilized sample in 100% D_2_O. Each HSQC spectrum was acquired with a total time of 93 s using the so-fast HSQC scheme. Amide hydrogen exchange rates were measured on a ^15^N-labeled sample containing 5% D_2_O and 95% H_2_O with a radiation-damping-based water inversion scheme described previously using an inter-scan delay of 2 s and 16 mixing times (20–300 ms)^27^.

### Analyses of CEST and RD data

When CEST or/and RD profiles were fitted to an exchange model, a standard χ^2^ based minimization procedure was employed. The χ^2^ is given by


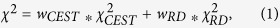



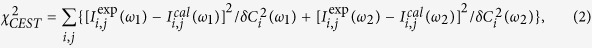



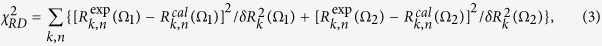



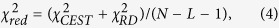


where I_i,j_^exp^(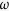
_m_) and I_i,j_^cal^(ω_m_) are the experimental and calculated intensities of the j^th^ CEST data point for the i^th^ residue at a weak rf field of ω_m_ respectively; R_k,n_^exp^(Ω_m_) and R_k,n_^cal^(Ω_m_) are the experimental and calculated relaxation rates of the n^th^ RD data point for the k^th^ residue at a static magnetic field of Ω_m_ respectively; δC_i_(ω_m_) and δR_i_(Ω_m_) are the errors of CEST intensities and relaxation rates for the i^th^ residue, respectively; w_CEST_ and w_RD_ are the weighting factors. If only CEST (RD) data are used, w_CEST_ (w_RD_) is set to 1 and w_RD_ (w_CEST_) is set zero. If both CEST and RD data are used, w_CEST_ is set to 1 and w_RD_ is set to T_C_/T_R_, where T_C_ and T_R_ are the total CEST and RD data points of a residue, respectively. The summation extends over all the data points for a given residue for individual fitting, while it extends over all the residues in a given protein region for global fitting. I_i,j_^cal^(ω_m_) and R_k,n_^cal^(Ω_m_) were calculated using the equations given in Supplementary Information, which were largely described previously^4^,^18^. N and L are the total experimental data points and fitting parameters, respectively. In the fitting, intrinsic transverse and longitudinal relaxation rates (R_2_ and R_1_) in all the states were assumed to be the same.

To estimate errors in the parameters obtained from the global fittings, ~80% of the available residues were selected randomly to form 50 sets of data and then each set was fitted. The error of each fitting parameter was obtained from the standard deviation of the 50 sets of results. Matlab scripts were used to fit the data, which are freely available upon request.

## Additional Information

**How to cite this article**: Yu, B. and Yang, D. Coexistence of multiple minor states of fatty acid binding protein and their functional relevance. *Sci. Rep.* **6**, 34171; doi: 10.1038/srep34171 (2016).

## Supplementary Material

Supplementary Information
