# Supplementary material for: Coexistence of multiple minor states of fatty acid binding protein and their functional relevance
Source: Sci Rep. 2016 Sep 28;6:34171. doi: 10.1038/srep34171 (PMC5039767; doi:10.1038/srep34171)
Supplement: Supplementary Information [file srep34171-s1.pdf]

Supplementary Information

Coexistence of multiple minor states of fatty acid binding protein and their functional relevance

Binhan Yu and Daiwen Yang*

Calculation of RD rates and CEST intensities for exchange models I and IV

The theoretical relaxation rate at a given CPMG field strength (ν_{CPMG}) was calculated by

$$R_n^{cal} = \frac{-\ln[M_{n\tau}(2)/M_0(2)]}{T_{CPMG}}, \qquad [1]$$

where T_{CPMG} = 4nτ and ν_{CPMG} =n/T_{CPMG}, T_{CPMG} is the total time of the CPMG period, τ is half of the delay between the centers of two successive 180° pulses, 2n is number of CPMG pulses; $M_{n\tau}(2)$ and $M_0(2)$ are the second element of magnetization vector $M_{n\tau}$ and M_0, respectively, $M_0 = [p_1\ p_2\ p_3]^T$ or $[p_1\ p_2\ p_3\ p_4]^T$ for 3-state or 4-state exchange, p_j is the population of state j (j=1, 2, 3, 4; which correspond to state I_1, N, I_2, and I_3 in scheme 1 in the main text); $M_{n\tau}$ is given by

$$M_{n\tau} = [\exp(-B\tau) * \exp(-B^*\tau) * \exp(-B^*\tau) * \exp(-B\tau)]^n * M_0, \qquad [2]$$

where B is the exchange matrix and B^* is the conjugate of B. For a 3-state exchange (model I), B is given by

$$B = \begin{bmatrix} R_{21} + k_{12} + i\Omega_1 & -k_{21} & 0 \\ -k_{12} & R_{22} + k_{21} + k_{23} + i\Omega_2 & -k_{32} \\ 0 & -k_{23} & R_{23} + k_{32} + i\Omega_3 \end{bmatrix}. \qquad [3]$$

For a 4-state exchange (model IV), B is given by

$$B = \begin{bmatrix} R_{21} + k_{12} + i\Omega_1 & -k_{21} & 0 & 0 \\ -k_{12} & R_{22} + k_{21} + k_{23} + k_{24} + i\Omega_2 & -k_{32} & -k_{42} \\ 0 & -k_{23} & R_{23} + k_{32} + i\Omega_3 & 0 \\ 0 & -k_{24} & 0 & R_{24} + k_{42} + i\Omega_4 \end{bmatrix}. \qquad [4]$$

In eqs. 3 and 4, R_{2j} and Ω_j are the transverse relaxation rate and resonant frequency (in radians per second) of a spin at state j, respectively; k_{jk} is the conversion rate from state j to state k; i = $\sqrt{-1}$.

The theoretical intensity of a spin at state N (or state 2) at a given weak rf field was calculated by

$$I^{cal} = M(6), \qquad [5]$$

where M(6) is the 6th element of magnetization vector M. M is given by

$$M = \exp(-At)M_0, \qquad [6]$$

where M_0 is the initial magnetization vector and equal to $[0\ 0\ p_1\ 0\ 0\ p_2\ 0\ 0\ p_3]^T$ for a 3-state exchange and $[0\ 0\ p_1\ 0\ 0\ p_2\ 0\ 0\ p_3\ 0\ 0\ p_4]^T$ for a 4-state exchange; t is the saturation time; A is an exchange matrix. For a 3-state model (model I), A is given by

$$A=\begin{bmatrix} R_{21}+k_{12} & -\Delta\omega_1 & \omega_y & -k_{21} & 0 & 0 & 0 & 0 & 0 \\ \Delta\omega_1 & R_{21}+k_{12} & -\omega_x & 0 & -k_{21} & 0 & 0 & 0 & 0 \\ -\omega_y & \omega_x & R_{11}+k_{12} & 0 & 0 & -k_{21} & 0 & 0 & 0 \\ -k_{12} & 0 & 0 & R_{22}+k_{21}+k_{23} & -\Delta\omega_2 & \omega_y & -k_{32} & 0 & 0 \\ 0 & -k_{12} & 0 & \Delta\omega_2 & R_{22}+k_{21}+k_{23} & -\omega_x & 0 & -k_{32} & 0 \\ 0 & 0 & -k_{12} & -\omega_y & \omega_x & R_{12}+k_{21}+k_{23} & 0 & 0 & -k_{32} \\ 0 & 0 & 0 & -k_{23} & 0 & 0 & R_{23}+k_{32} & -\Delta\omega_3 & \omega_y \\ 0 & 0 & 0 & 0 & -k_{23} & 0 & \Delta\omega_3 & R_{23}+k_{32} & -\omega_x \\ 0 & 0 & 0 & 0 & 0 & -k_{23} & -\omega_y & \omega_x & R_{13}+k_{32} \end{bmatrix}. \quad [7]$$

For a 4-state model (model IV), A is given by

$$A=\begin{bmatrix} R_{21}+k_{12} & -\Delta\omega_1 & \omega_y & -k_{21} & 0 & 0 & 0 & 0 & 0 & 0 & 0 & 0 \\ \Delta\omega_1 & R_{21}+k_{12} & -\omega_x & 0 & -k_{21} & 0 & 0 & 0 & 0 & 0 & 0 & 0 \\ -\omega_y & \omega_x & R_{11}+k_{12} & 0 & 0 & -k_{21} & 0 & 0 & 0 & 0 & 0 & 0 \\ -k_{12} & 0 & 0 & R_{22}+k_{21}+k_{23}+k_{24} & -\Delta\omega_2 & \omega_y & -k_{32} & 0 & 0 & -k_{42} & 0 & 0 \\ 0 & -k_{12} & 0 & \Delta\omega_2 & R_{22}+k_{21}+k_{23}+k_{24} & \omega_x & 0 & -k_{32} & 0 & 0 & -k_{42} & 0 \\ 0 & 0 & -k_{12} & -\omega_y & \omega_x & R_{12}+k_{21}+k_{23}+k_{24} & 0 & 0 & -k_{32} & 0 & 0 & -k_{42} \\ 0 & 0 & 0 & -k_{23} & 0 & 0 & R_{23}+k_{32} & -\Delta\omega_3 & \omega_y & 0 & 0 & 0 \\ 0 & 0 & 0 & 0 & -k_{23} & 0 & \Delta\omega_3 & R_{23}+k_{32} & -\omega_x & 0 & 0 & 0 \\ 0 & 0 & 0 & 0 & 0 & -k_{23} & -\omega_y & \omega_x & R_{13}+k_{32} & 0 & 0 & 0 \\ 0 & 0 & 0 & -k_{24} & 0 & 0 & 0 & 0 & 0 & R_{24}+k_{42} & -\Delta\omega_4 & \omega_y \\ 0 & 0 & 0 & 0 & -k_{24} & 0 & 0 & 0 & 0 & -\Delta\omega_4 & R_{24}+k_{42} & -\omega_x \\ 0 & 0 & 0 & 0 & 0 & -k_{24} & 0 & 0 & 0 & -\omega_y & \omega_x & R_{14}+k_{42} \end{bmatrix}. \quad [8]$$

In eqs. 7 and 8, $\Delta\omega_j = \Omega_j - \omega_{rf}$, where ω_{rf} is the angular frequency of the weak rf field applied in CEST; ω_x and ω_y are the x and y components of the rf field strength (in radians per second); R_{1j} is the longitudinal relaxation rate of a spin at state j.

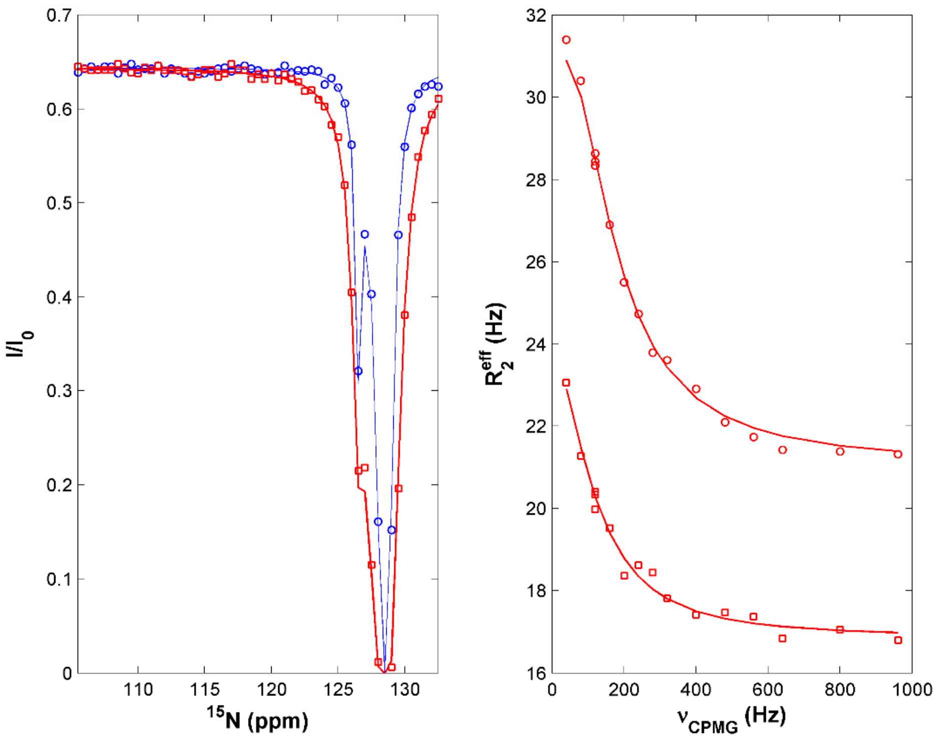

Figure S1. Representative experimental and calculated CEST (left panel) and RD (right panel) profiles described well by model I (Q42). Experimental CEST points recorded with rf field strengths of 13.6 and 27.2 Hz are indicated by ○ and □, respectively. RD data points recorded on 800 and 500 MHz are indicated by ○ and □, respectively. The best fits obtained with model I are solid lines.

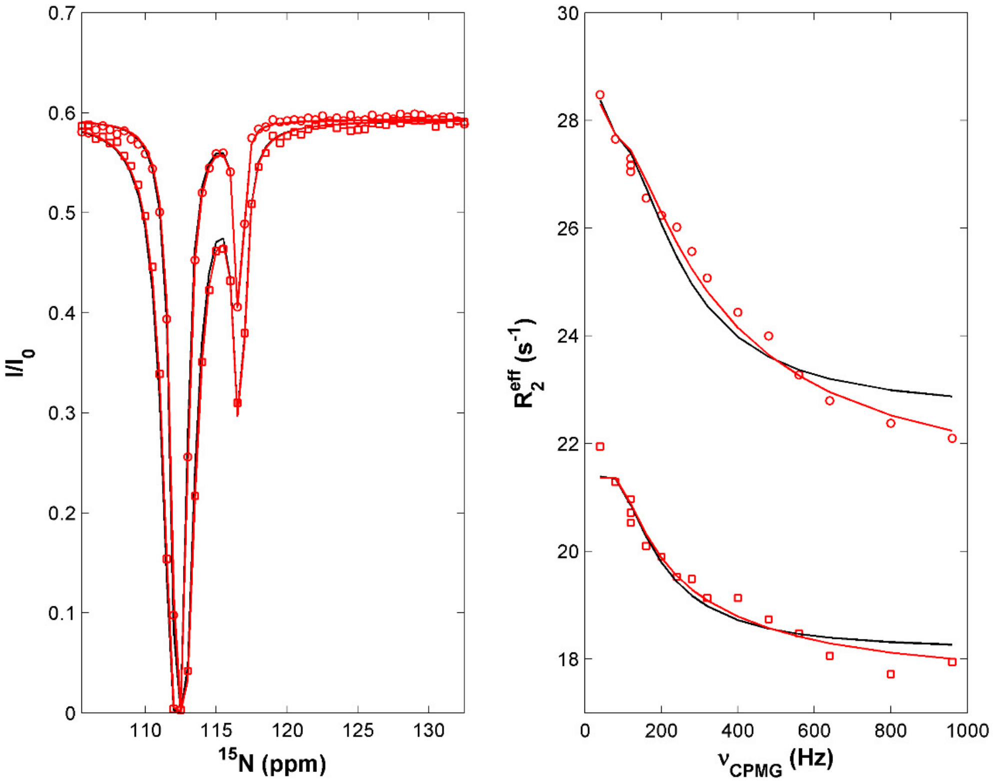

Figure S2. Representative experimental and calculated CEST (left panel) and RD (right panel) profiles described well by model IV rather than by model I (F55). Experimental CEST points recorded with rf field strengths of 13.6 and 27.2 Hz are indicated by ○ and □, respectively. RD data points recorded on 800 and 500 MHz are indicated by ○ and □, respectively. The best fits obtained with model I (3-state) are black solid lines, while the fits obtained with model IV (4-state) are red lines.

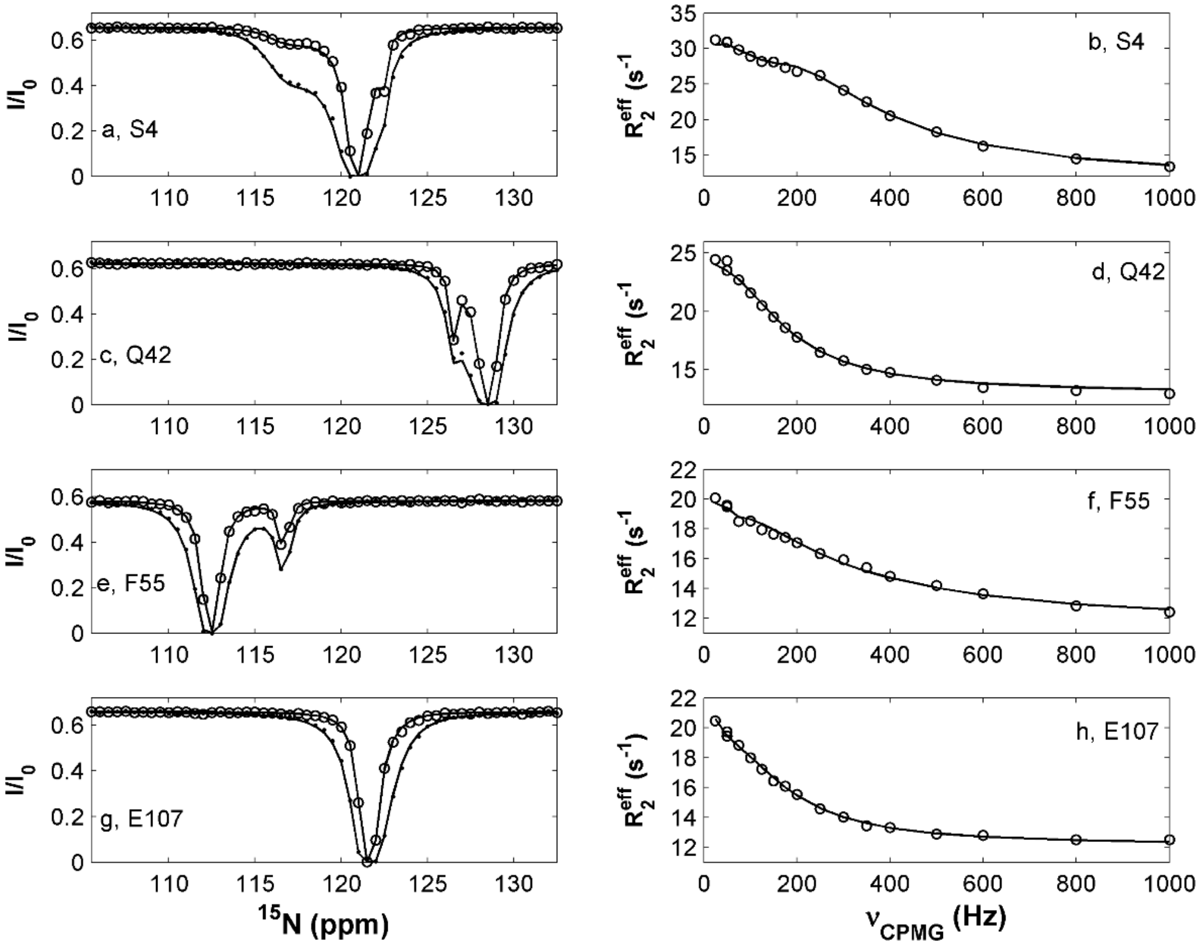

Figure S3. Representative CEST (a, c, e, and g) and RD (b, d, f, and h) profiles recorded at 0.7 mM hIFABP. The experimental CEST data at rf field fields of 13.6 and 27.2 Hz are indicated by "o" and "●", respectively. The experimental RD data at 800 are indicated by "o". The solid lines are best fits obtained with model I (a, b, c, d, g, and h) and model IV (e and f). Note that the R_2^{eff} values shown here were recorded with the continuous wave decoupling CPMG scheme, which are significantly smaller than those shown in Figure 1 that were acquired with the relaxation compensated scheme.

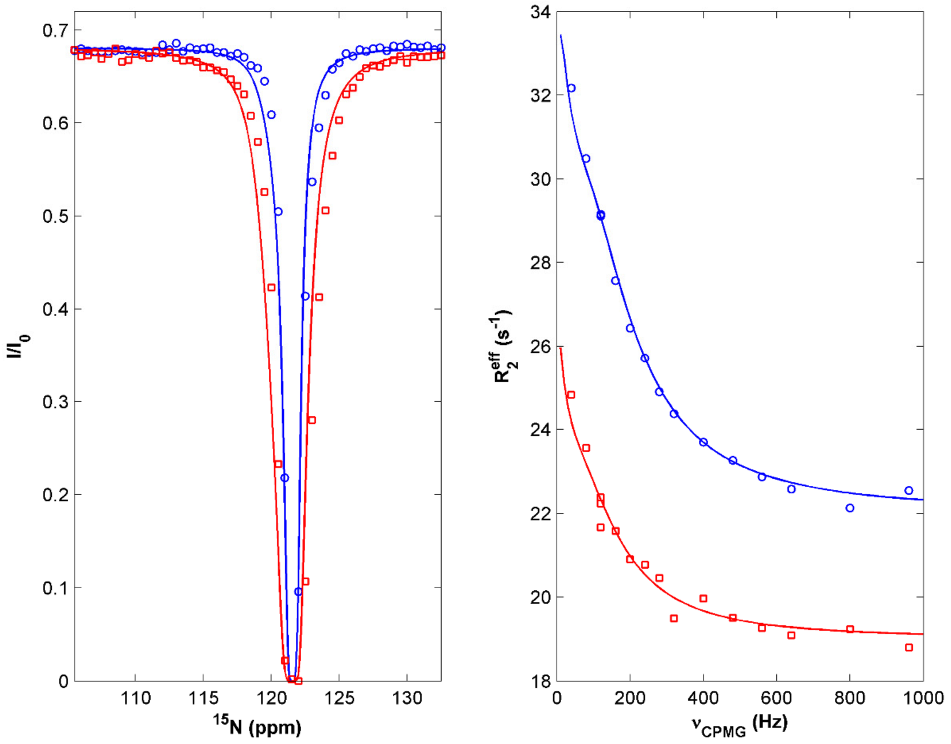

Figure S4. Experimental and calculated CEST (left panel) and RD (right panel) profiles of E107. Experimental CEST points recorded with rf field strengths of 13.6 and 27.2 Hz are indicated by ○ and □, respectively. RD data points recorded on 800 and 500 MHz are indicated by ○ and □, respectively. The solid lines were calculated with model I by assuming $\nu(I_1) - \nu(N) = -0.41$ ppm and $\nu(I_2) - \nu(N) = -1.83$ ppm.

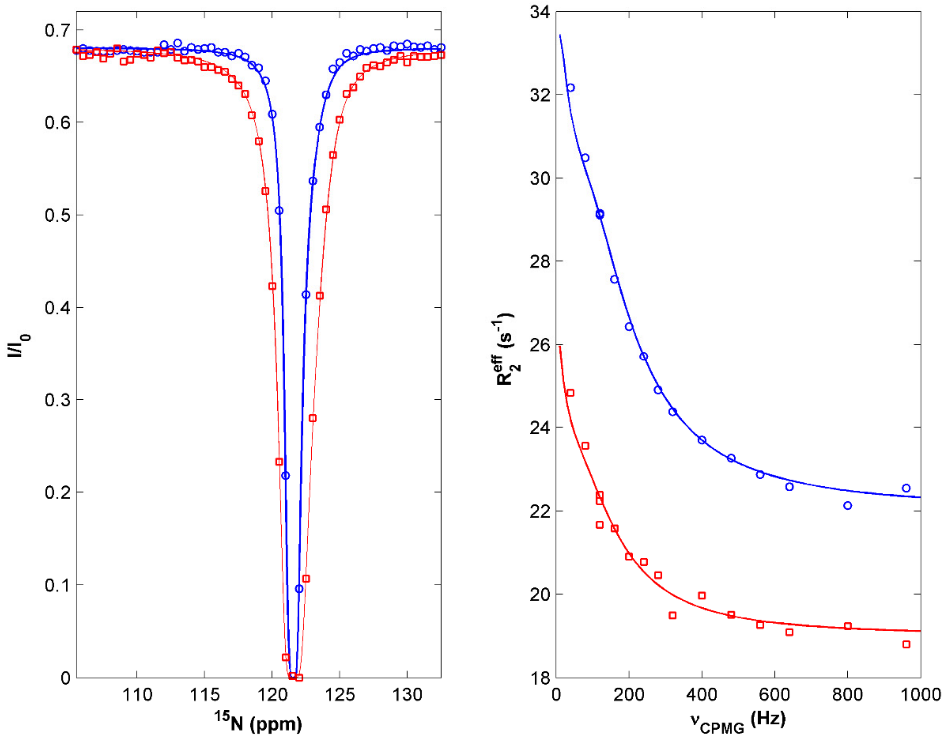

Figure S5. The experimental data used are the same as those shown in Fig. S4. However, the solid lines were calculated with model I by assuming $\nu(I_1) - \nu(N) = -0.41$ ppm and $\nu(I_2) - \nu(N) = +1.83$ ppm.

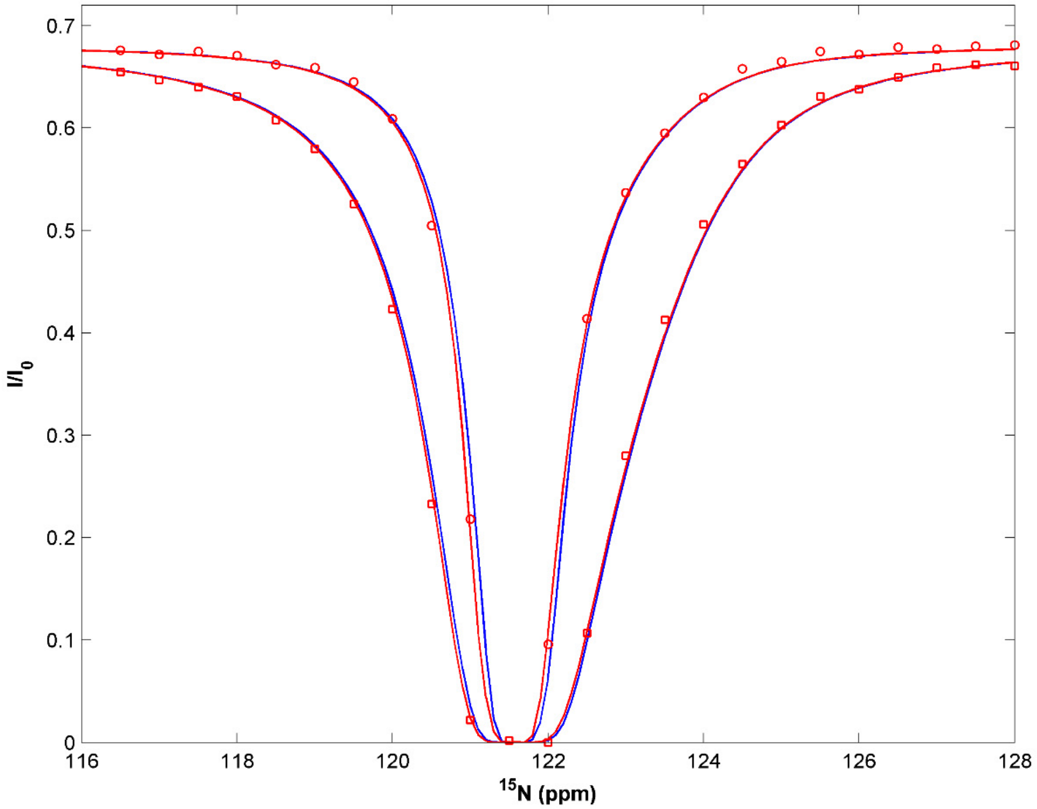

Figure S6. The experimental data used are the same as those shown in Fig. S4. Only the region from 116-128 ppm are displayed for better view. The red lines were calculated by assuming $\nu(I_1) - \nu(N) = -0.41$ ppm and $\nu(I_2) - \nu(N) = 1.83$ ppm*, while the blue lines were obtained by assuming* $\nu(I_1) - \nu(N) = +0.41$ ppm and $\nu(I_2) - \nu(N) = 1.83$ ppm.

Table S1. ^{15}N chemical shifts of major state (N) and minor states (I_1, I_2, and I_3) and amide hydrogen exchange rates (k_{obs}) and protection factors

Res	^{15}N chemical shifts (ppm)					Amide hydrogen exchange	
	N	I_1	I_2	I_3	U[a]	$k_{obs}(s^{-1})$[b]	P (k_{rc}/k_{ex})[c]
F2*	113.63	111.84	116.45		119.13	0.6	13.3
D3	119.26	118.35	120.65		122.39	0.4	20.0
S4*	120.99	122.35	116.24		116.69	0.3	71.3
T5*	116.24	115.02	114.19		116.05	1.2	19.6
W6*	128.75	129.19	125.75		123.48	$1.20x10^{-4}$	$7.10x10^{4}$
K7	123.47	123.86	120.98		122.10	$1.44x10^{-4}$	$6.80x10^{4}$
V8*	128.39	126.62	127.58		121.34	-	-
D9	128.19	128.61	127.38		124.65	$7.20x10^{-4}$	$6.98x10^{3}$
R10	112.92	112.66	113.65		121.91	$1.26x10^{-3}$	$8.72x10^{3}$
S11	113.75	112.78	113.12		117.74	3.3	16.3
E12*	122.62	121.25	123.18		122.85	$1.84x10^{-2}$	$4.63x10^{2}$
Y14	121.14	121.79	119.71		120.55	1.7	9.1
D15	118.59	-	-		123.00	-	-
K16*	119.69	120.68	119.12		121.55	0.7	11.9
F17	121.43	n	n		121.30	$2.23x10^{-4}$	$4.71x10^{4}$
M18	118.21	118.39	117.55		122.14	$1.55x10^{-4}$	$1.00x10^{5}$
E19	120.13	n	n		122.24	$2.51x10^{-4}$	$2.19x10^{4}$
K20	123.12	n	n		122.43	$6.14x10^{-4}$	$1.45x10^{4}$
M21	115.43	n	n		121.31	$6.07x10^{-4}$	$2.94x10^{4}$
G22	108.30	108.85	107.75		110.14	0.3	110.7
V23	121.53	120.84	120.96		120.18	0.4	10.2
N24*	126.50	127.65	127.69		123.01	6.0	5.1
I25*	121.19	122.75	122.34		121.16	4.7	1.1
V26*	121.67	120.31	122.09		124.68	0.7	2.3
K27	119.32	n	n		125.80	2.3	4.0
R28	119.52	n	n		122.67	3.4	6.4
K29	119.50	118.79	119.05		122.86	6.6	3.2
L30	119.71	119.28	119.06		123.18	1.4	3.4
A31	120.15	121.30	121.02		124.74	1.9	4.5
A32*	117.17	119.57	116.31		123.04	3.4	4.1
H33*	116.88	115.56	117.90		117.92	5.0	2.2
D34*	119.08	120.77	118.31		121.75	3.0	3.2
N35*	119.17	120.69	118.08		118.95	3.9	7.2
L36*	119.55	120.84	120.37		122.64	0.3	25.4
K37*	126.41	128.31	125.69		121.79	0.01-0.1	78-780
L38*	123.06	125.75	125.55		123.26	$2.20x10^{-4}$	$2.18x10^{4}$
T39*	121.29	117.83	122.42		114.69	$3.13x10^{-4}$	$2.32x10^{4}$
I40	128.58	129.17	126.47		123.27	$2.42x10^{-3}$	$1.69x10^{3}$
T41*	121.31	122.97	119.88		118.14	$4.56x10^{-4}$	$1.52x10^{4}$
Q42*	128.53	126.46	126.85		122.80	0.3	84.0
E43	128.65	128.05	127.83		122.47	0.2	34.0
G44	117.63	-	-		109.79	-	-
N45	113.18	112.45	114.48		118.74	9.9	6.4
K46	120.89	n	n	n	121.57	$8.87x10^{-3}$	$2.97x10^{3}$

F47*	126.35	124.45	127.05	125.30	121.53	$1.31x10^{-2}$	$8.01x10^{2}$
T48*	115.81	119.18	116.25	113.76	116.22	$3.85x10^{-4}$	$3.51x10^{4}$
V49	127.81	128.12	127.27	126.24	123.15	$2.21x10^{-4}$	$1.98x10^{4}$
K50*	128.28	123.79	127.42	126.59	125.76	$3.06x10^{-4}$	$2.99x10^{4}$
E51*	127.07	127.90	129.33	124.69	122.03	$2.87x10^{-3}$	$1.96x10^{3}$
S52*	120.40	119.69	121.73	120.26	117.11	-	-
S53*	121.42	115.37	122.12	119.73	118.23	0.4	161.6
A54*	120.59	126.83	119.64	122.58	125.72	5.5	5.0
F55*	112.39	116.68	111.77	114.36	119.43	1.6	5.0
R56*	114.46	118.17	113.09	116.84	123.31	0.9	21.2
N57	120.44	-	-	-	120.41	-	-
I58*	118.54	117.01	117.03	120.59	121.11	0.3	17.9
E59*	124.54	126.00	123.25	126.86	124.93	1.1	2.3
V60*	127.55	125.01	128.90	123.15	121.58	0.01-0.1	19.5-195
V61	125.72	126.03	125.53	123.62	124.43	$6.14x10^{-3}$	$3.25x10^{2}$
F62*	121.44	123.32	121.11	122.71	124.55	$3.09x10^{-5}$	$1.87x10^{5}$
E63	120.25	120.66	120.63	118.84	122.61	$6.72x10^{-3}$	$7.30x10^{2}$
L64*	125.14	123.20	125.26	122.95	123.10	$1.49x10^{-3}$	$1.73x10^{3}$
G65	108.76	109.78	108.59	110.08	109.57	$5.15x10^{-3}$	$3.08x10^{3}$
V66*	122.47	121.25	122.49	120.72	120.48	$4.38x10^{-5}$	$9.32x10^{4}$
T67*	129.48	128.14	128.67		118.05	7.3	1.1
F68	127.05	n	n		122.60	$1.38x10^{-4}$	$9.14x10^{4}$
N69	117.23	117.70	116.55		120.52	0.3	163.6
Y70*	122.32	125.87	123.26		120.71	0.2	77.5
N71	121.02	121.81	123.41		121.00	1.0	48.0
L72*	121.99	120.70	122.84		122.57	$3.53x10^{-3}$	$2.15x10^{3}$
G75	108.22	107.93	109.04		109.54	2.6	6.5
T76	119.99	n	n		113.92	2.0	8.7
E77*	129.02	127.99	129.57		123.05	0.3	22.7
L78	126.09	125.70	125.29		123.04	$1.54x10^{-4}$	$1.67x10^{4}$
R79	119.40	n	n		122.12	$5.48x10^{-6}$	$2.95x10^{6}$
G80	116.32	115.77	115.56		110.77	$4.45x10^{-5}$	$9.61x10^{5}$
T81	107.05	107.91	106.47		113.75	$3.21x10^{-5}$	$5.43x10^{5}$
W82	120.37	119.79	121.63		123.36	-	-
S83	115.84	n	n		116.33	$5.29x10^{-6}$	$4.76x10^{6}$
L84	125.63	n	n		124.07	0.2	36.2
E85	127.85	128.43	127.30		121.60	0.2	13.2
G86	117.63	-	-		109.79	-	-
N87*	122.87	121.32	122.21		118.74	9.6	6.5
K88	118.32	n	n		121.65	$2.33x10^{-3}$	$1.13x10^{4}$
L89*	123.76	121.59	122.83		123.02	$<5x10^{-6}$	$>3.6x10^{6}$
I90	125.39	n	n		121.35	$<5x10^{-6}$	$>1.2x10^{7}$
G91	122.27	121.90	121.46		113.06	$<5x10^{-6}$	$>5.2x10^{6}$
K92	128.45	128.82	127.59		120.87	$3.34x10^{-4}$	$5.59x10^{4}$
F93	121.93	n	n		121.52	$1.01x10^{-4}$	$7.53x10^{4}$
K94	119.69	n	n		123.53	$1.83x10^{-5}$	$7.92x10^{5}$
R95	122.40	122.86	123.32		122.67	0.4	54.7
T96	116.23	115.77	117.23		115.56	1.1	17.7
D97	121.02	n	n		122.67	0.4	27.5
N98	117.04	n	n		119.35	0.5	56.4
G99	108.15	n	n		109.26	0.3	179.3

N100	119.63	n	n	118.57	$9.75x10^{-3}$	$6.48x10^{3}$
E101	120.36	n	n	121.07	0.01-0.1	89.3-893
L102	124.63	125.25	125.35	122.88	$3.57x10^{-5}$	$7.22x10^{4}$
N103	124.71	124.33	124.09	119.58	$1.98x10^{-4}$	$1.33x10^{5}$
T104	118.71	118.71	117.66	114.68	$2.09x10^{-4}$	$1.177x10^{5}$
V105	127.27	n	n	123.02	$4.45x10^{-5}$	$9.83x10^{4}$
R106	124.58	n	n	125.70	$4.08x10^{-5}$	$2.95x10^{5}$
E107	121.53	121.12	123.36	122.51	$4.53x10^{-5}$	$1.57x10^{5}$
I108	124.86	-	-	121.57	-	-
I109	130.26	n	n	124.72	$5.14x10^{-4}$	$2.95x10^{3}$
G110	119.37	n	n	112.61	11.8	1.3
D111	124.86	-	-	120.11	-	-
E112	118.68	n	n	121.05	$1.34x10^{-4}$	$2.11x10^{4}$
L113	123.96	n	n	123.06	$1.58x10^{-5}$	$1.63x10^{5}$
V114	128.30	128.63	127.59	121.15	$<5x10^{-6}$	$>3.4x10^{5}$
Q115	130.73	n	n	124.81	$9.19x10^{-5}$	$1.25x10^{5}$
T116*	122.04	120.92	120.53	115.34	$1.22x10^{-4}$	$1.53x10^{5}$
Y117	126.24	125.77	125.32	122.86	$1.01 x10^{-4}$	$1.16 x10^{5}$
V118	120.17	120.79	120.89	122.75	$7.09x10^{-5}$	$4.37x10^{4}$
Y119	128.22	n	n	124.63	$9.83x10^{-5}$	$5.47x10^{4}$
E120	126.26	n	n	123.50	0.2	24
G121	127.72	n	n	109.96	0.01-0.1	182.3-1823
V122	123.70	n	n	120.30	$2.56x10^{-4}$	$1.59x10^{4}$
E123	126.68	n	n	125.27	$2.27x10^{-2}$	$1.36x10^{2}$
A124	126.38	125.83	127.05	125.20	$3.04x10^{-4}$	$3.22x10^{4}$
K125	116.66	117.39	115.96	120.65	$7.37x10^{-4}$	$1.71x10^{4}$
R126*	120.50	121.69	119.64	122.43	$2.06x10^{-4}$	$1.06x10^{5}$
I127	123.59	123.17	122.96	121.94	$1.17x10^{-4}$	$3.65x10^{4}$
F128	126.90	n	n	124.41	$1.83x10^{-4}$	$2.56x10^{4}$
K129	118.80	118.42	120.38	123.66	$1.97x10^{-4}$	$7.35x10^{4}$
K130	123.72	123.20	125.54	122.29	$5.48x10^{-3}$	$3.03x10^{3}$
D131*	130.32	129.16	131.01	na	$7.16x10^{-3}$	$1.28x10^{3}$

n: No RD dispersion was observed on the 800 MHz spectrometer, indicating the chemical shifts in the native and intermediate states are very similar.

-: due to peak overlap or weak peak, the data are not available.

na: the prediction value is not available.

*: CEST profiles displayed two obvious dips (>80 Hz) and as well in RD profiles R_{ex} > 2 s^{-1} on 500 MHz NMR.

[a:] The shifts in the unfolded state were predicted using the ncIDP predictor tool (http://nmr.chem.rug.nl/ncIDP/).
[b:] For the exchange rates larger than 0.1 s^{-1}, they were measured by the amide hydrogen exchange method in 95% H_2O and 5% D_2O. For the rates smaller than 0.01 s^{-1}, they were measured by the H-D exchange method. For those between 0.01 and 0.1 s^{-1}, they were estimated based on the dead time (180 s) of the H-D exchange experiment and the lower limit of the amide hydrogen exchange method. The H-D exchange data were recorded for 5.5 hours at pH 7.1, 30 °C. So the exchange rates could not be measured accurately if they are smaller than $5x10^{-6}$ s^{-1}.

[c:] k_{rc} was predicted using an online software tool (http://sblab.sastra.edu/cintx.html).
